# Lactoferrin-Anchored Tannylated Mesoporous Silica Nanomaterials for Enhanced Osteo-Differentiation Ability

**DOI:** 10.3390/pharmaceutics13010030

**Published:** 2020-12-26

**Authors:** Sung Hyun Noh, Han-Saem Jo, Somang Choi, Hee Gyeong Song, Hak-Jun Kim, Keung Nyun Kim, Sung Eun Kim, Kyeongsoon Park

**Affiliations:** 1 Department of Neurosurgery, National Health Insurance Service Ilsan Hospital, #100, Ilsan-ro, Ilsan-donggu, Gyeonggi-do, Goyang-si 10444, Korea; ulove07@nhimc.or.kr; 2 Department of Systems Biotechnology, Chung-Ang University, Gyeonggi-do, Anseong-si 17546, Korea; luchiatkfkd@naver.com (H.-S.J.); island6231@gmail.com (H.G.S.); 3 Department of Orthopedic Surgery and Rare Diseases Institute, Korea University Guro Hospital, #148, Gurodong-ro, Guro-gu, Seoul 08308, Korea; chlthakd1029@naver.com (S.C.); dakjul@korea.ac.kr (H.-J.K.); 4 Department of Neurosurgery, Spine and Spinal Cord Institute, Severance Hospital, Yonsei University College of Medicine, #50, Yonsei-ro, Seodaemun-gu, Seoul 03722, Korea

**Keywords:** mesoporous silica nanomaterials, lactoferrin, adipose-derived stem cells, osteo-differentiation ability

## Abstract

In the present study, we created lactoferrin-anchored mesoporous silica nanomaterials with absorbed tannic acid (LF/TA-MSNs) and evaluated the effect of these LF/TA-MSNs on the in vitro osteo-differentiation ability of adipose-derived stem cells (ADSCs) by testing alkaline phosphatase (ALP) level, calcium accumulation, and expression of osteo-differentiation-specific genes, including osteocalcin (*OCN*) and osteopontin (*OPN*). Both bare MSNs and LF/TA-MSNs exhibited round nano-particle structures. The LF/TA-MSNs demonstrated prolonged LF release for up to 28 days. Treatment of ADSCs with LF (50 μg)/TA-MSNs resulted in markedly higher ALP level and calcium accumulation compared to treatment with LF (10 μg)/TA-MSNs or bare MSNs. Furthermore, LF (50 μg)/TA-MSNs remarkably increased mRNA levels of osteo-differentiation-specific genes, including *OCN* and *OPN*, compared to MSNs or LF (10 μg)/TA-MSNs. Together, these data suggest that the ability of LF/TA-MSNs to enhance osteo-differentiation of ADSCs make them a possible nanovehicle for bone healing and bone regeneration in patients with bone defect or disease.

## 1. Introduction

Spinal fusion is an end-stage treatment to relieve the symptoms of lower-back pain by prohibiting motion in the affected segment [1]. Fusion surgeries are performed on the cervical, thoracic, and lumbar spine for treatment of numerous morbidities, including trauma, deformity, and degeneration [2,3]. Currently, several types of grafts have been used for spine fusion surgery. Iliac bone grafts (IBGs, autobone grafts) are widely used to improve bone fusion in spinal fusion surgery. IBGs are the gold standard grafting approach for spinal fusion surgery due to their excellent fusion rate and fusion time [4]. However, IBGs also have major disadvantages, such as pain at the donor site, nerve damage and inflammation, and prolonged surgical time, which limit their use in osteoporosis and metabolic diseases [5,6,7,8]. Allografts, acquired from either a cadaver or a living donor, come in one of three subtypes: fresh-frozen, freeze-dried, or demineralized bone matrix (DBM) [4]. However, their use is limited by certain restrictions, such as lack of osteogenic properties, limited risk of hepatitis B virus (HBV) or HCV infection, and possible disease transmission [3,9,10].

To overcome these drawbacks, new materials have been developed to enhance bone regeneration. For example, exosomes released from all cell types are natural nanovesicles and act as a shuttle of therapeutic molecules with pro-regenerative properties [11,12]. Additionally, they have a great potential in critical bone defect healing, neo-angiogenesis, and bone formation [13,14]. Calcium silicates (CaSi) are biointeractive materials and widely used for the regeneration of mineralized tissues such as bone and dentine [15]. They release biologically active ions including OH^−^ and Ca^2+^ ions and provide an excellent stimulus for bone formation [16,17]. Recently, biodegradable polymers have been used alone or combined with devices for bone repair or surgical dressings [18]. Additionally, highly porous polymeric scaffolds doped with bioactive CaSi and/or exosomes have been developed to provide adequate thermal-mechanical properties and to improve osteogenic commitment of stem cells, resulting in promising materials as space fillers [19,20]. However, despite their enhanced bone regeneration ability in vivo, the development of new materials with osteoconductive potency still remains a challenging field.

Lactoferrin (LF) is a powerful antiviral, anti-inflammatory, and antimicrobial substance contained only in cow colostrum and human colostrum [21]. It is an iron-bound protein that plays an important role in compounding and liberating iron [22]. In addition, LF increases immunity and activity of macrophages by interacting with various immune cells and helps to strengthen immune secretory organs by synthesizing with various proteins [23]. Furthermore, LF has capacity as an osteogenic growth factor. Previous reports have shown that LF treatment not only increased osteoblast proliferation, but also promoted osteoblast differentiation [24,25]. Other studies have demonstrated that LF not only advanced new bone formation in calvarial defects, but also enhanced bone mineral density in osteoporosis [26,27]. Our previous studies showed that LF-coated hydroxyapatite (HAp) nanocrystals and LF-immobilized microspheres increased osteogenic differentiation in rabbit ADSCs [28,29]. Recently, our team fabricated LF-coated nanodiamonds (NDs) and confirmed their osteogenic activity by showing the increment of alkaline phosphatase (ALP) and calcium levels [30]. More recently, Choi et al. [31] reported that LF nanoparticles not only significantly suppressed levels of pro-inflammatory factors in IL-1β induced cells, but also enhanced tendon restoration in Achilles tendinitis rodents. However, although LF itself is a promising protein for osteogenic differentiation and tendon healing, the efficacy of protein drug has not been effective than we expected due to its short half-life in the blood without help of delivery vehicle [31,32].

Tannic acid (TA), a specific form of a hydrolysable natural polyphenol, is found in many plant-based foods and beverages, such as green tea, coffee, red wine, hazelnuts, and walnuts [33]. Recently, it has been revealed that TA can function as both an organic and inorganic specimen due to the existence of hydroxyl groups and galloyl groups [34,35]. TA is known to directly interact with several kinds of biomacromolecules (DNA, gelatin, collagen, albumin, chitosan, thrombin, and enzymes) due to its electrostatic, hydrogen bonding, and hydrophobic interaction abilities [36].

Recently, mesoporous silica nanomaterials (MSNs) have been attracting considerable attention for their potential in biomedical applications due to their superior biocompatibility and biodegradability compared to other inorganic nanomaterials [37,38]. Moreover, MSNs have been reported in the past few years as an important topic of research in bone tissue engineering owing to their high surface area, large pore size, and easily modifiable surface [39,40]. Due to these and other benefits, they have garnered attention as a promising drug nanovehicle that possesses not only a high drug loading ability, but also the ability to deliver time-dependent drug release.

In this study, to achieve long-term delivery LF using MSNs and to enhance osteoinductive effects on stem cells, the surface of MSNs as delivery nanovehicle were modified with TA, and LF was further anchored on tannylated MSNs (TA-MSNs). Then, we evaluated the ability of LF-anchored tannylated MSNs (LF/TA-MSNs) to induce osteo-differentiation of ADSCs in vitro.

## 2. Materials and Methods

### 2.1. MSNs Modified by TA and LF

For anchoring of lactoferrin (human LF, Sigma-Aldrich, St. Louis, MO, USA), mesoporous silica nanomaterials (MSNs, Sigma-Aldrich) were modified by tannic acid (TA, Sigma-Aldrich). First, MSNs (10 mg) were added to phosphate-buffered saline (PBS) solution (pH 7.4) containing dissolved TA (50 μg/mL), and the mixture was lightly shaken overnight at room temperature (RT). After that, TA-laden MSNs were rinsed 2 times with distilled water (DW) and lyophilized for 2 days. The amount of residual TA in the PBS solution was analyzed to confirm the loading amount of TA on MSNs. TA-laden MSNs are hereafter referred to as TA-MSNs. For anchoring of LF (10 or 50 μg/mL) on the MSN surface, TA-MSNs (10 mg/mL) and LF (10 or 50 μg/mL) was added into PBS solution, followed by incubation for 24 h. Next, all samples were washed 3 times with DW at 3000 rpm and 4 °C for 10 min using a Smart R17 Centrifuge (Hanil Science Industrial, Incheon, Korea), followed by freeze-drying for 2 days. To assess the loading amount of LF, the supernatant was collected following anchoring of LF to TA-MSNs and analyzed with a Pierce Bicinchoninic Acid (BCA) Protein Assay Kit (Thermo Fisher Scientific, Rockford, IL, USA) following the manufacturer’s protocol. The LF (10 μg)-anchored TA-MSNs and LF (50 μg)-anchored TA-MSNs were designated as LF (10 μg)/TA-MSNs and LF (50 μg)/TA-MSNs, respectively.

### 2.2. Characterization

For morphological analysis using a transmission electron microscope (TEM), each sample including MSNs, TA-MSNs, LF (10 μg)/TA-MSNs, and LF (50 μg)/TA-MSNs was added in ethanol and then dispersed using an ultra-sonicator (Hwashin Tech Co., Seoul, Korea) for 1 h. Each group was carefully moved to the TEM plate, and the shape of each MSN was observed with the TEM (JEM-F200, JEOL Ltd., Tokyo, Japan) at the Yonsei Center for Research Facilities. For particle sizes, size distributions, and surface charge assays, each substance (0.1 mg) was re-dispersed in DW using an ultra-sonicator at 4 °C. The dispersed groups were analyzed by a dynamic light scattering (DLS) device (Malvern Instruments, Malvern, UK) using a Helium-Neon laser (633 nm). To analyze the surface chemical compositions of MSNs, TA-MSNs, LF (10 μg)/TA-MSNs, and LF (50 μg)/TA-MSNs, an ESCALAB 250 X-ray Photoelectron Spectrometer (XPS) with an ultra-high vacuum (1 × 10^−9^ bar) (Thermo Fisher Scientific Inc., Waltham, UK) supplemented with an Al K_α_ X-ray source (1486.6 eV photons) was used to measure the C1s, N1s, O1s, and Si2p of each group. The surface area, pore volume, and pore size of the naked and modified MSNs were characterized in accordance with the Brunauer, Emmet, Teller (BET) and Barrett, Joyner, Halenda (BJH) techniques using a nitrogen adsorption instrument (Autosorb-iQ 2ST/MP, Quantachrome Instruments Co., Boynton Beach, FL, USA).

### 2.3. LF Release from TA-MSNs

The in vitro amount of LF release from the LF/TA-MSNs was determined as follows. Ten mg of each sample was dispersed onto a membrane bag containing 1 mL of PBS (pH 7.4), and the dialysis membranes were placed into a 15 mL tube with PBS (5 mL, pH 7.4), followed by mild agitation in a warm water bath (37 °C) that oscillated at 100 times/min. The PBS was replenished at predetermined time intervals (1, 5, 9, 12 h, 1, 3, 5, 7, 14, 21, and 28 days). The released amount of LF was calculated using a Pierce BCA Protein Assay Kit according to the manufacturer’s instructions and a Multimode Reader (Varioskan™, Thermo Scientific, Waltham, MA, USA) at 562 nm. The number of samples assessed per group was 4. The lactoferrin release experiment was repeated three times.

### 2.4. Cytotoxicity Assays

Prior to assessing the effect of LF/TA-MSNs on cellular activity, we conducted cytotoxicity tests on each group. For this, 1 × 10^4^ adipose-derived stem cells (ADSCs, Lonza Group Ltd., Basel, Switzerland) were seeded in 96-well plates and cultivated in DMEM at 37 °C overnight. After that, cells were washed three times with PBS and exposed to one of the three sample of MSNs (100 μg/mL). At 1 and 3 days, DMEM was removed, and fresh PBS was added to wash the cells. Then, reagent from the Cell Counting Kit-8 (CCK-8, Dojindo Molecular Technologies, Inc. Rockville, MD, USA) was added to each well, and cells were cultured for 1 h in the dark at 37 °C. Absorbance at 450 nm was measured using a Multimode Reader. The control group consisted of untreated ADSCs. Cytotoxicity was evaluated as the percentage of viable cells versus the control group. The number of samples assessed per group was 4. All experiments for each time period were repeated three times.

### 2.5. Cellular Uptake

To verify cellular uptake, MSNs were modified with β-cyclodextrin (β-CD, Tokyo Chemical Industry Co., Ltd., Tokyo, Japan) and fluorescein isothiocyanate (FITC, Thermo Fisher Scientific, USA). This β-CD (1 mg·mL^−1^) was dissolved in DW, followed by addition of FITC (100 μg), and the solution was gently stirred under dark conditions for 4 h. After incubation, TA-MSNs (1 mg) were added to the DW solution and incubated at RT in the dark overnight. At the end of the incubation period, the product was centrifuged using a Micro Refrigerated Centrifuge at 3000 rpm for 10 min at 4 °C and rinsed with DW, followed by lyophilization for 2 days using a freeze dryer (FD8508, IlShinBioBase Co., Ltd. Gyeonggido, Korea). Cells were seeded at a density of 1 × 10^4^ cells per confocal dish (SPL Life Sciences, Pocheon-si, Gyeonggi-do, Korea) and allowed to incubate for 24 h. Next, cells were exposed to DMEM with FITC/β-CD-MSNs (100 μg) and incubated for 4 h. Cells then were rinsed with PBS and fixed with 4% paraformaldehyde for 30 min, followed by addition of 4-6-diamidino-2-phenylindole (DAPI, Thermo Fisher Scientific, USA). The mixture was incubated for 30 min at RT. Finally, samples were observed using a confocal laser scanning microscope (CLSM, LSM700, Zeiss, Jena, Germany).

### 2.6. Initial Osteo-Differentiation Marker

To estimate the initial osteo-differentiation ability of LF/TA-MSNs of ADSCs, quantification of ALP level was performed at each endpoint. The ADSCs (1 × 10^5^ cells/well) were cultured in DMEM medium containing 10% FBS and 1% antibiotics and then plated in 24-well plates and exposed to MSNs and LF (10 μg or 50 μg)/TA-MSNs at a density of 100 μg/mL. After being exposed to each sample for 3 or 9 days, cells were rinsed with PBS and lysed with lysis buffer (1× RIPA buffer). After centrifugation at 13,500 rpm for 10 min, the supernatant of the harvested cell lysates. The protein concentration of the supernatant was measured by Bradford assay using bovine serum albumin as a standard. Then, the lysate (60 μL) was blended with *P*-nitrophenyl phosphate (Sigma-Aldrich, USA), followed by incubation at 37 °C for 30 min. Then, 1 N NaOH (500 μL) was added to the solution to stop the reaction. Absorbance was recorded at 405 nm with a Multimode Reader. ALP activity was analyzed by measuring the release of *P*-nitrophenyl from *P*-nitrophenyl phosphate and also determined by the following equation. ALP activity (μM/min/μg) = the released *P*-nitrophenyl/protein concentration of cell extract × microliters of cell extract/min. All experiments for each time period were repeated three times.

### 2.7. Late Osteo-Differentiation Marker

The calcium content of the ADSCs was evaluated at pre-set time periods to serve as a late osteo-differentiation marker. The number of seeded cells and sample concentrations in the calcium contents experiment were the same as those in the ALP experiments described above. At the end of pre-designated time intervals (7 and 21 days), cells were exposed to 0.5N HCl (500 μL) and incubated with stirring at 37 °C overnight. After that, cells were incubated with a calcium standard solution and reagent solutions containing both *O*-cresolphthalein complexone (Sigma-Aldrich) and 8-hydroxy-quindine (Sigma-Aldrich) for 1 min, followed by addition of 2-amino-2-methyl-1-propanol (Sigma-Aldrich) buffer. The full resultant solution was blended for 15 min. Afterward, the terminal solution was cautiously shifted to 96-well plates, and absorbance was measured with a Multimode Reader at 575 nm. All experiments for each time period were repeated three times.

### 2.8. Quantification of Osteo-Differentiation Specific Genes

To further confirm the osteogenic ability of MSNs with and without LF, the mRNA levels of osteo-differentiation-specific gene markers, such as osteocalcin (OCN) and osteopontin (OPN), were investigated using real-time PCR. The ADSCs (1 × 10^5^ cells) were cultured with one of the three types of MSNs (100 μg/mL). After 21 days of culture, RNeasy Plus Mini Kit was used to measure the total ribonucleic acid (RNA) in cells. After quantification of total RNA levels, reverse-transcription of total RNA (1 μg) was performed to generate cDNA using AccuPower RT PreMix. The primer sequences of the *OCN* and *OPN* genes are described in the Supplementary Materials (Table S1). PCR amplification and real-time PCR were carried out using an ABI7300 Real-Time Thermal Cycler (Applied Biosystems, Foster, CA, USA). The *OCN* and *OPN* genes were normalized using glyceraldehyde 3-phophate dehydrogenase (GAPDH). All experiments for each time point were repeated three times.

### 2.9. Statistical Analysis

All data are presented as mean ± standard deviation. Statistical analysis was conducted using a one-way Anova in SigmaPlot (SPPSS Inc., Systat Software, Ver. 12, Chicago, IL, USA) by Holm-Sidak t-test for multiple comparisons. *p* values were compared among all MSN groups. *p* values less than 0.05 and 0.01 were regarded as statistically significant.

## 3. Results

### 3.1. Characterization

**Figure 1:**
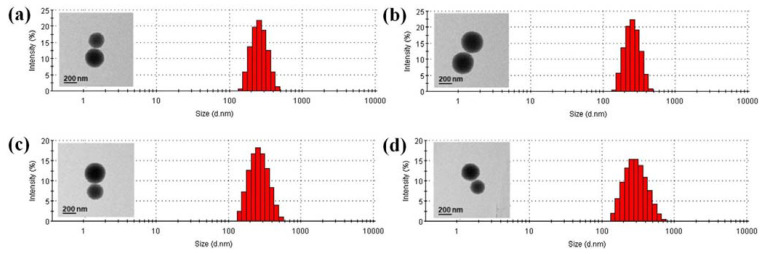
Size distribution curves of (**a**) MSNs, (**b**) TA-MSNs, (**c**) LF (10 μg)/TA-MSNs, and (**d**) LF (50 μg)/TA-MSNs. Inset: TEM images of each group. Scale bar: 200 nm.

The laden amount and efficiency of TA binding to TA-MSNs were 48.40 ± 0.01 μg and 96.8 ± 0.02%, respectively. The confirmed loading amount (efficiency) of LF was 9.9 ± 0.2 μg (98.80 ± 1.6%) for LF (10 μg)/TA-MSNs and 48.60 ± 0.5 μg (97.10 ± 1.0%) for LF (50 μg)/TA-MSNs. The shapes of MSNs with and without anchorage to TA and/or LF were assessed by TEM (Figure 1). Each MSN group observed by TEM had a spherical morphology and nano-range. The average size distribution and polydispersity index were 264.30 ± 65.39 nm and 0.228 for MSNs, 247.10 ± 62.55 nm and 0.040 for TA-MSNs, 264.10 ± 81.61 nm and 0.205 for LF (10 μg)/TA-MSNs, and 276.40 ± 108.8 nm and 0.153 for LF (50 μg)/TA-MSNs, respectively. The certified zeta potential rates of the MSNs, TA-MSNs, LF (10 μg)/TA-MSNs, and LF (50 μg)/TA-MSNs were −10.8 ± 1.2, −18.1 ± 1.2, −10.5 ± 0.9, and −7.6 ± 1.4 mV, respectively. As shown in Figure S1, after surface modification of MSNs with TA molecules, the zeta potential values decreased due to the negative charge of TA molecules. When TA-MSNs were further modified with different amounts of LF, the zeta potential values of LF/TA-MSNs increased depending on the amounts of LF, suggesting that the positively charged LF protein molecules were successfully modified on the surfaces of TA-MSNs.

The XPS valuation was used to assess to the surface chemical compositions of MSNs, TA-MSNs, LF (10 μg)/TA-MSNs, and LF (50 μg)/TA-MSNs (Table 1 and Figure S2). As shown in Figure S2, the Si2p, C1s, and O1s peaks in all groups were detected at 103 eV, 284 eV, and 532 eV, respectively. The N1s peaks in two LF/TA-MSNs groups were observed at approximately 398.5~402 eV, but not in MSNs and TA-MSNs groups. MSNs modified by TA had an estimated increase in O1s component from 58.58% to 62.82% and a decline in Si2p component from 36.68% to 32.77% compared with unmodified MSNs. The N1s and O1s components of the LF (10 μg)/TA-MSNs increased by 0.47% and decreased by 2.03%, respectively, compared with TA-MSNs. The LF (50 μg)/TA-MSNs had an increase in N1s component from 0.47% to 0.72% compared with LF (10 μg)/TA-MSNs.

As shown in Table 2, the BET surface areas and pore volumes of LF-anchored MSNs or TA-coated MSNs were lower than those of naked MSNs.

### 3.2. Release Pattern of LF

**Figure 2:**
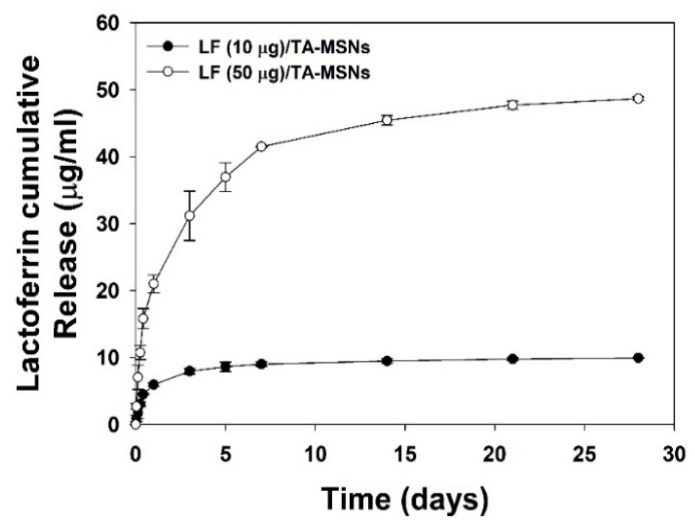
In vitro cumulative release patterns of LF from the LF (10 μg)/TA-MSNs and LF (50 μg)/TA-MSNs (*n* = 4 per group).

The release patterns of LF from the LF (10 μg)/TA-MSNs and LF (50 μg)/TA-MSNs over 28 days are exhibited in Figure 2. The released LF was 5.93 ± 0.27 μg (60.08 ± 2.79%) for LF (10 μg)/TA-MSNs and 20.99 ± 1.33 μg (43.24 ± 2.75%) for LF (50 μg)/TA-MSNs at 1 day. At 28 days, the LF (10 μg)/TA-MSNs and LF (50 μg)/TA-MSNs had released 9.90 ± 0.2 μg (99.98 ± 0.88%) and 48.59 ± 0.27 μg (99.99 ± 0.56%) of LF, respectively.

### 3.3. In Vitro Cytotoxicity and Cellular Uptake Analyses

**Figure 3:**
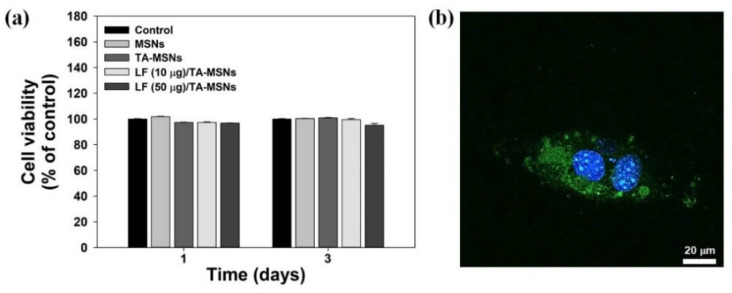
(**a**) Results of cytotoxicity assays following treatment of ADSCs with MSNs, TA-MSNs, LF (10 μg)/TA-MSNs, or LF (50 μg)/TA-MSNs at 1 and 3 days. (**b**) In vitro intracellular tracking of FITC-tagged MSNs after 4 h of culture. Green color indicates the intracellular uptake of FITC-tagged MSNs particles into cells, while blue color represents DAPI-stained nuclei of cells. Scale bar: 20 μm.

Figure 3a shows the results of the cytotoxicity analyses for each treatment group at 1 and 3 days of culture with ADSCs. Cell survival after treatment with each MSN sample was maintained at greater than 98% of the control group viability for 3 days. Therefore, no cytotoxicity was seen in ADSCs after treatment with each MSN, confirming in vitro safety. Figure 3b displays the intracellular tracking of FITC-tagged MSNs acquired by laser confocal scanning microscopy (LCSM). After 4 h of treatment, FITC-tagged MSNs or some of their aggregates were obviously observed into the cytoplasm of the ADSCs.

### 3.4. Evaluation of Initial and Late Osteo-Differentiation Markers in ADSCs Treated with MSNs

**Figure 4:**
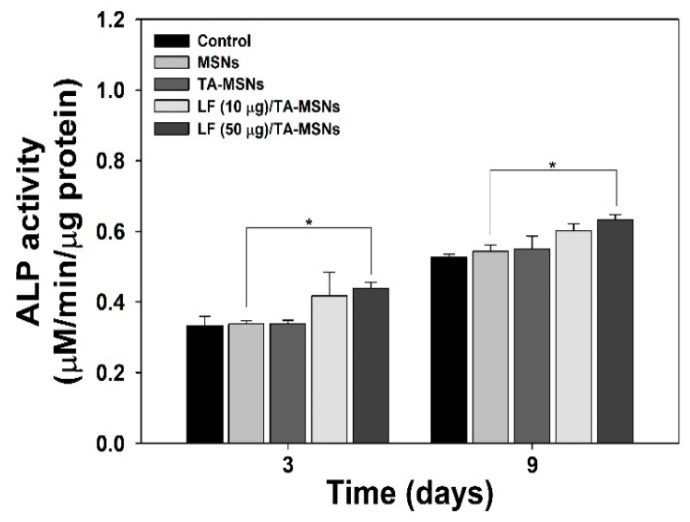
ALP level in ADSCs cultured with MSNs, TA-MSNs, LF (10 μg)/TA-MSNs, or LF (50 μg)/TA-MSNs after 3 and 9 days of inoculation (*n* = 4 per group). * *p* < 0.05.

ALP level was assessed to establish the initial osteo-differentiation ability of ADSCs treated with various MSNs. As shown in Figure 4, ALP level in ADSCs treated with MSNs, TA-MSNs, LF (10 μg)/TA-MSNs, or LF (50 μg)/TA-MSNs increased over the cultivation period of 9 days. On day 3, there was a significant difference in the level of ALP in ADSCs treated with LF (50 μg)/TA-MSNs compared to those treated with bare MSNs (* *p* < 0.05). Moreover, the level of ALP in ADSCs grown with LF (50 μg)/TA-MSNs after 9 days was remarkably higher than that grown with bare MSNs (* *p* < 0.05). Meanwhile, there were no significant differences in expression level of ALP between ADSCs treated with LF (10 μg)/TA-MSNs and those treated with LF (50 μg)/TA-MSNs at 3 and 9 days.

**Figure 5:**
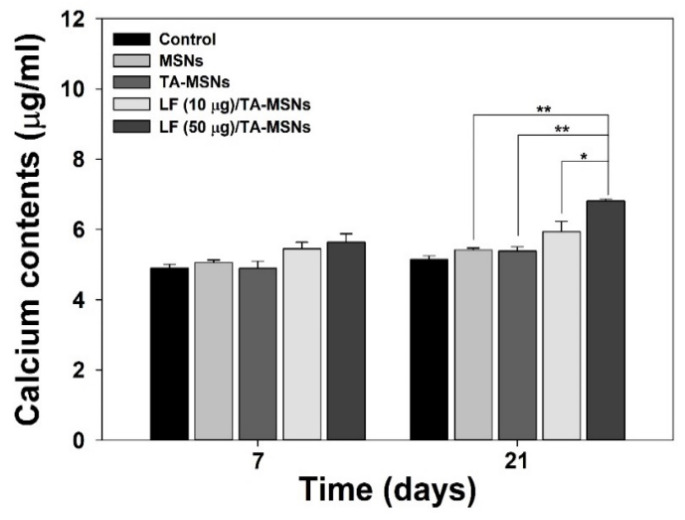
Calcium accumulation in adipose-derived stem cells (ADSCs) cultured with bare MSNs, TA-MSNs, LF (10 μg)/TA-MSNs, or LF (50 μg)/TA-MSNs after 7 and 21 days of incubation (*n* = 4 per group). * *p* < 0.05 and ** *p* < 0.01.

To analyze the late osteo-differentiation ability of ADSCs treated with MSNs, we investigated calcium accumulation. As shown in Figure 5, the quantity of calcium accumulated in ADSCs treated with each MSN group gradually increased in a time-dependent manner. On day 7, there were no significant differences in total calcium accumulation in ADSCs between MSN treatment groups. On day 14, the quantity of calcium in ADSCs cultivated with LF (50 μg)/TA-MSNs was higher than that of ADSCs cultured with bare MSNs or TA-MSNs (* *p* < 0.05). On day 21, calcium accumulation in ADSCs cultured with LF (50 μg)/TA-MSNs was markedly greater than in ADSCs grown with bare MSNs or TA-MSNs (** *p* < 0.01). Moreover, a significant difference in the total amount of calcium deposited by cells was exhibited between LF (50 μg)/TA-MSN and LF (10 μg)/TA-MSN treatment groups (* *p* < 0.05).

### 3.5. Osteo-Differentiation-Specific Genes

**Figure 6:**
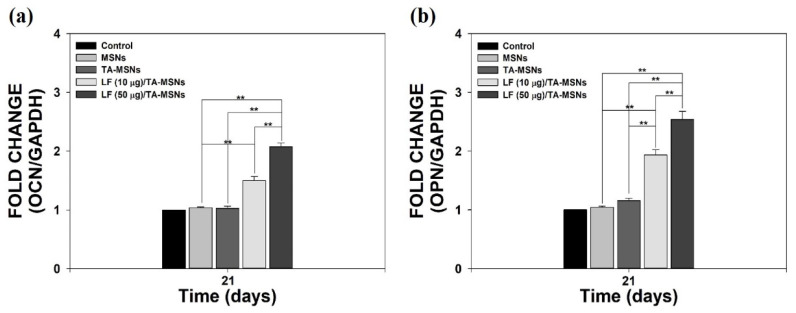
The mRNA levels of (**a**) *OCN* and (**b**) *OPN* in ADSCs cultured with bare MSNs, TA-MSNs, LF (10 μg)/TA-MSNs, or LF (50 μg)/TA-MSNs after 7 and 21 days of incubation (*n* = 4 per group). ** *p* < 0.01.

To further verify whether the osteo-differentiation ability of ADSCs was changed by treatment with LF/TA-MSNs, we calculated the expression levels of osteo-differentiation-specific indicators, including *OCN* and *OPN* genes, with real-time PCR after 21 days of incubation (Figure 6a,b). *OCN* and *OPN* gene expression in ADSCs treated with TA-MSNs containing LF (10 μg) was much higher than in those treated with bare MSNs (** *p* < 0.01). *OCN* and *OPN* levels were remarkably greater on day 21 in ADSCs cultured with LF (50 μg)/TA-MSNs than in cells cultivated with bare MSNs or TA-MSNs (** *p* < 0.01). *OCN* and *OPN* expression levels were significantly different between cells treated with LF (50 μg)/TA-MSNs and those treated with LF (10 μg)/TA-MSNs (** *p* < 0.01).

## 4. Discussion

The development of new materials for bone tissue regeneration is still challenging, although numerous biomaterials have been proposed for this aim. Among them, silica or silicates are attractive inorganic materials as bone tissue scaffolds. Despite silica or silicates act as an excellent stimulus for bone formation [16,17], these basic materials showed the mere effect on bone formation. In this study, we developed new type of LF/TA-MSNs to provide long-term release of LF from nanoparticles and to endow osteoconductive effects of silica nanoparticles. The goal of the present study was to assess whether LF/TA-MSNs could enhance the osteo-differentiation ability of ADSCs. To prepare LF/TA-MSNs, LF was anchored to MSN surfaces covered with a TA coating. Each MSN group was observed by TEM, and the DLS ranged from 260 to 300 nm. Moreover, XPS was employed to investigate the surface chemical makeup of MSNs with TA and/or LF and compare it with that of bare MSNs. After coating with TA, MSNs exhibited both an increase in O1s content and a decrease in Si2p content compared to bare MSNs. After anchoring of LF onto TA-MSN surfaces, N1s content newly appeared. Lee et al. [35] showed that TA adsorbed by a PCL substrate increased O1s content compared to that of the PCL substrate alone. In addition, TA adsorbed by a PCL substrate after immobilizing BMP-2 exhibited an increased N1s content. These prior outcomes were consistent with the results of this study, suggesting that we achieved successful TA and/or LF attachment on MSN surfaces. TA is a representative molecule with powerful affinity for a diverse array of proteins, such as proline-rich proteins [41]. As formerly noted, BMP-2 absorbed by a TA-PCL substrate led to sustained release of BMP-2 compared to that of the PCL substrate alone [35]. Coincidental with these outcomes, LF/TA-MSNs showed sustained release of LF for up to 28 days. This result indicates that LF/TA-MSNs nanoparticles may achieve the long-term delivery of LF. Although there are some limitations to explain the uptake mechanism of MSNs, FITC-tagged MSNs nanoparticles were internalized and distributed into cytoplasm of ADSCs, indicating that the use of MSNs may have advantage for effective delivery of LF molecules into cells for a long period.

Previous studies have shown that LF is an osteoinductive factors that can promote osteogenic differentiation of various cells, including osteoblasts, C2C12, and ADSCs [42,43]. Takayama et al. [44] demonstrated that LF mixed with a collagen membrane promoted an increase in ALP level and expression of *OCN* in human osteosarcoma (MG-63) cells. Previous studies demonstrated that oral administration of LF not only improved bone formation and bone consolidation in a distraction osteogenesis animal model, but also enhanced bone mineral density (BMD) and biomechanical strength in an ovariectomized animal model [45,46].

Owing to the superior properties of LF, as described above, we investigated the ability of LF/TA-MSNs to enhance osteo-differentiation of adipose-derived stem cells (ADSCs) via examination of an initial osteo-differentiation marker, a late osteo-differentiation marker, and osteo-differentiation-specific genes. Osteo-differentiation is divided into three steps: proliferation, matrix formation, and mineralization [47]. Alkaline phosphatase, a well-known cell membrane-associated enzyme, has previously shown early expression coincident with osteoblast differentiation and has been widely suggested to be an early osteoblastic differentiation marker [28,29,47,48]. In addition, increased ALP level has been shown to be associated with matrix formation in osteoblasts, prior to onset of mineralization [49]. The ALP level of ADSCs treated with MSNs, TA-MSNs, LF (10 μg)/TA-MSNs, or LF (50 μg)/TA-MSNs was examined at 3 and 9 days of incubation. The ALP level of ADSCs cultured with each MSN group increased in a time-dependent manner. However, ALP level was significantly different between ADSCs treated with LF (50 μg)/TA-MSNs and those treated with bare MSNs at 3 days of culture. Moreover, ALP level was significantly higher in ADSCs treated with LF (50 μg)/TA-MSNs than those treated with bare MSN at 9 days of incubation. Calcium content has been typically used as a late osteo-differentiation marker [28,29,30,35]. At 7 days of culture, there were no significant differences in calcium deposition between any MSN treatment groups. However, significant differences in calcium deposition were observed between ADSCs treated with TA-MSNs in the presence of LF and those treated with bare MSNs after 21 days of incubation. Moreover, the amount of calcium deposited in ADSCs cultured with LF (50 μg)/TA-MSNs was remarkably higher than that in ADSCs cultured with LF (10 μg)/TA-MSNs. The results shown by Kim et al. [28,29,30,47] demonstrated that treatment with LF combined with different types of substrates, such as granules, nanocrystals, NDs, and titanium, increased ALP level and calcium accumulation. As reported previously, LF treatment increased ALP level and calcium deposition in ADSCs in a dose-dependent manner [43]. These previous results were consonant with our outcomes, indicating that treatment with LF is correlated with increases in initial osteo-differentiation markers and late osteo-differentiation markers, such as ALP level and calcium content.

To further inquire into the effect of MSNs on the osteo-differentiation of ADSCs, we performed real-time PCR to estimate the mRNA levels of osteo-differentiation-specific genes (*OCN* and *OPN*) in ADSCs treated with each MSN group after days 21 of inoculation. *OCN* and *OPN*, which are known to regulate osteoblast differentiation, show increased expression in osteoblast-like cells [28,29]. *OCN*, an element expressed and secreted only by osteoblasts, has been used as a serum marker of bone formation [48,50]. *OPN*, a versatile extracellular matrix-related glycoprotein, is important in parathyroid hormone (PTH) regulation [51]. *OCN* and *OPN* mRNA levels in ADSCs cultured with LF (50 μg)/TA-MSNs were notably greater than those in cells cultured with bare MSNs. Furthermore, *OCN* and *OPN* mRNA expression levels were significantly different between ADSCs treated with LF (50 μg)/TA-MSNs and those treated with LF (10 μg)/TA-MSNs. Previously, our research group demonstrated that the mRNA levels of *OCN* and *OPN* in ADSCs cultivated with LF-microparticles or LF-hydroxyapatite nanocrystals were notably increased compared to the levels in control cells cultured in the absence of LF. These outcomes indicate that LF directly up-regulates expression of osteo-differentiation-specific genes, including *OCN* and *OPN*.

As biologically active molecules, LF promotes proliferation and differentiation of various cells including osteoblasts, C2C12, and ADSCs [42,43]. Additionally, it inhibits osteoclastogenesis by reducing the number of osteoclasts that can actively resorb bone [52]. Owing to these positive effects of LF, the use of LF in bone tissue regeneration is of great interest owing However, due to its poor bioavailability in vivo, a nanomaterials-based strategy has been developed to improve the biological activities of LF. In this study, LF/TA-MSNs are able to release LF for a long period and internalized well into ADSCs cells, leading to the enhanced osteo-differentiation of ADSCs. Till now, it was reported that the osteo-differentiation mechanism of LF is associated with the mitogenic effects of LF in osteoblasts [53]. However, additional investigation for osteo-differentiation mechanism of LF/TA-MSNs should be required. More importantly, LF/TA-MSNs are powder-type materials and thus readily combined with synthetic polymers to improve mechanical property as well as osteogenic potency of composite scaffolds. In near future, we hope at LF/TA-MSNs or composite scaffolds doped with LF/TA-MSNs can be applied as temporary space fillers and bond grafts for bone regeneration in orthopedic and dental surgery.

## 5. Conclusions

The aim of this study was to investigate whether the osteo-differentiation ability of adipose-derived stem cells (ADSCs) could be enhanced by treatment with LF/TA-MSNs. LF/TA-MSNs were prepared as follows. LF was anchored to MSN surfaces following MSN absorption of TA. The LF/TA-MSNs demonstrated prolonged release of LF. The LF/TA-MSNs promoted osteo-differentiation of ADSCs by significantly increasing ALP expression level and calcium accumulation compared with ADSCs cultured with bare MSNs. Moreover, LF/TA-MSNs promoted osteo-differentiation of ADSCs to significantly increase the mRNA levels of OCN and OPN (osteo-differentiation-specific genes) compared with those cultivated with bare MSNs. Accordingly, these LF/TA-MSNs may be able to be used to accelerate bone healing and regeneration of bone fractures and disorders.

## Figures and Tables

**Table 1:** Elemental compositions of bare and modified MSNs by XPS analysis.

Elements Groups	C1s (%)	N1s (%)	O1s (%)	Si2p (%)	Total (%)
MSNs	4.74	-	58.58	36.68	100
TA-MSNs	4.40	-	62.82	32.77	100
LF (10 μg)/TA-MSNs	3.81	0.47	60.79	34.93	100
LF (50 μg)/TA-MSNs	3.79	0.72	60.20	35.29	100

**Table 2:** Adsorption and desorption parameters of all MSN groups.

Sample	BET Surface Area (m^2^/g)	Total Pore Volume at BJH Desorption (cc/g)	Average Pore Volume at BJH Desorption (nm)
MSNs	287.248	2.474	3.446
TA-MSNs	267.011	2.283	3.287
LF (10 μg)/TA-MSNs	211.103	2.047	3.016
LF (50 μg)/TA-MSNs	202.087	1.943	2.825

## Data Availability

The data presented in this study are available in supplementary materials.

## Supplementary Materials

The following are available online at https://www.mdpi.com/1999-4923/13/1/30/s1, Table S1: The primer sequences of OCN and OPN (the osteo-differentiation specified genes), Figure S1: Zeta potential curves of MSNs, TA-MSNs, LF (10 μg)/TA-MSNs, and LF (50 μg)/TA-MSNs. Sky blue-colored arrow heads indicate the changes of zeta potential values after surface modification at each step, Figure S2: XPS spectra of MSNs, TA-MSNs, LF (10 μg)/TA-MSNs, and LF (50 μg)/TA-MSNs. Magnified results showed N1s peaks.
