# Supplementary material for: Lactoferrin-Anchored Tannylated Mesoporous Silica Nanomaterials for Enhanced Osteo-Differentiation Ability
Source: Pharmaceutics. 2020 Dec 26;13(1):30. doi: 10.3390/pharmaceutics13010030 (PMC7823981; doi:10.3390/pharmaceutics13010030)
Supplement: Supplementary file 1 [file pharmaceutics-13-00030-s001.pdf]

pharmaceutics

MDPI

Supplementary Materials: Lactoferrin-Anchored Tannylated Mesoporous Silica Nanomaterials for Enhanced Osteo-Differentiation Ability

Sung Hyun Noh, Han-Saem Jo, Somang Choi, Hee Gyeong Song, Hak-Jun Kim, Keung Nyun Kim, Sung Eun Kim and Kyeongsoon Park

Table S1. Primer sequences of OCN and OPN (the osteo-differentiation-specific genes)

Gene	Primer (forward)	Primer (reverse)
OCN	TTG GTG CAC ACC TAG CAG AC	ACC TTA TTG CCC TGC TT
OPN	GAG GGC TTG GTT GTC AGC	CAA TTC TCA TGG TAG TGA GTT TTC C

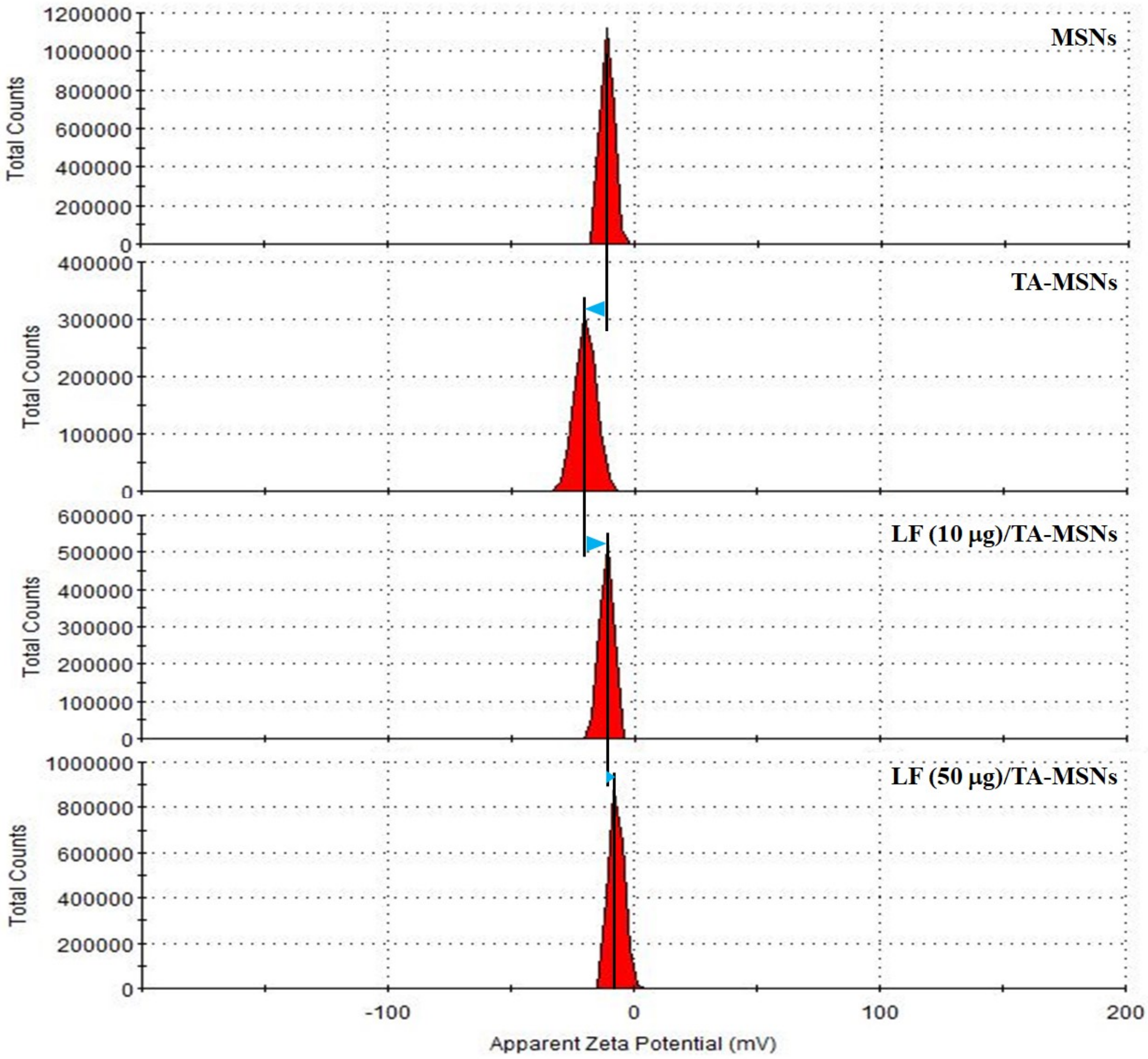

Figure S1. Zeta potential curves of MSNs, TA-MSNs, LF (10 µg)/TA-MSNs, and LF (50 µg)/TA-MSNs. Sky blue-colored arrow heads indicate the changes of zeta potential values after surface modification at each step.

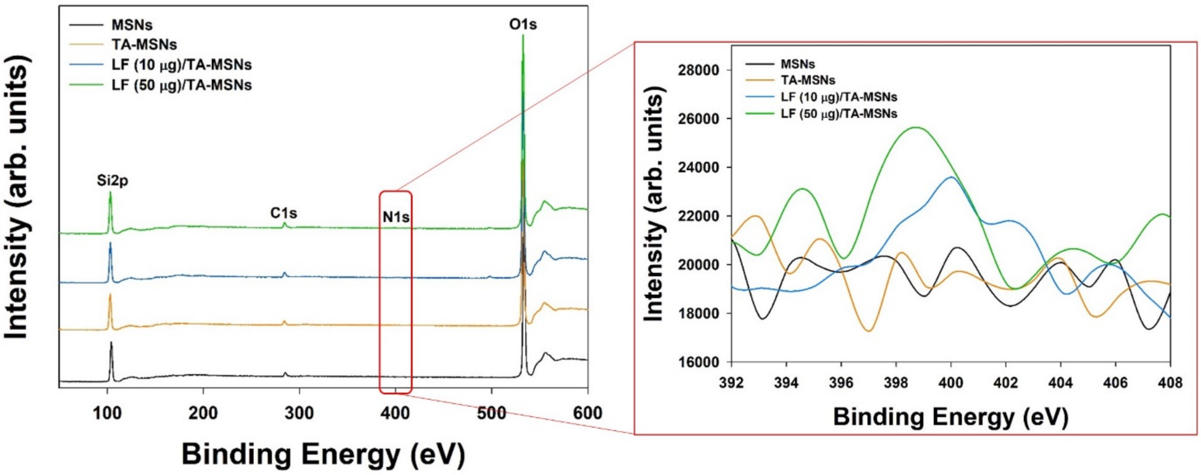

Figure S2. XPS spectra of MSNs, TA-MSNs, LF (10 μg)/TA-MSNs, and LF (50 μg)/TA-MSNs. Magnified results showed N1s peaks.
